# Obstructive sleep apnea syndrome: complaints and housing characteristics in a population in the United States

**DOI:** 10.1590/1516-3180.2013.1314451

**Published:** 2013-08-01

**Authors:** Khalil Ansarin, Leyla Sahebi, Siamak Sabur

**Affiliations:** I MD. Internist, Pulmonologist and Chairman of Tuberculosis and Lung Disease Research Center, Tabriz University of Medical Sciences, Tabriz, Iran.; II MSc. Doctoral Student of Epidemiology, Tuberculosis and Lung Disease Research Center, Tabriz University of Medical Science, Tabriz, Iran.; III MD, PhD. Assistant Professor of Clinical Epidemiology and Medicine, Department of Clinical Epidemiology, School of Dentistry, Shahid Beheshti University of Medical Sciences, Tehran, Iran.

**Keywords:** Sleep apnea, obstructive, Housing, Nutrition surveys, Snoring, United States, Apnéia do sono tipo obstrutiva, Habitação, Inquéritos nutricionais, Ronco, Estados Unidos

## Abstract

**CONTEXT AND OBJECTIVE:**

Obstructive sleep apnea syndrome (OSAS) is one of the leading causes of morbidity and mortality in adults. Early detection of the disorder and discovery of risk factors through standardized questionnaires will lead to reduction of the OSAS burden. The main aim of this study was to estimate the prevalence of OSAS symptoms and examine their association with housing characteristics.

**DESIGN AND SETTING:**

Cross-sectional study at a medical school.

**METHODS:**

Demographic, housing and body measurement data on 5,545 individuals aged 16 years and over of various races were selected from the National Health and Nutrition Examination Survey. We analyzed the probability of OSAS based on habitual snoring combined with daytime sleepiness and/or witnessed apnea. Univariate and multiple linear regression were used.

**RESULTS:**

9.8% of the men and 6.9% of the women reported symptoms suggestive of OSAS (habitual snoring, daytime sleepiness and/or apnea). The following prevalences of symptoms were found among males and females respectively: frequent snoring 35.1%, 22.3%, excessive daytime sleepiness 6.4%, 3.4% and frequent apnea 14.9%, 20.6%. Using multiple linear regression, OSAS symptoms were correlated with gender, age, body mass index (BMI), marital status and education. Regarding housing characteristics, mildew or musty smell and pets in the environment were associated with a high probability of OSAS.

**CONCLUSION:**

OSAS symptoms were more prevalent than in developing countries. The environment was an important risk factor, but environmental factors are easier to control and manage than other variables like BMI or socioeconomic status.

## RESUMO

**CONTEXTO E OBJETIVO:**

Síndrome de apneia obstrutiva do sono (SAOS) é uma das principais causas de morbidade e mortalidade em adultos. Detecção precoce da doença e descoberta de fatores de risco com questionários padronizados levarão a redução dos danos por SAOS. O principal objetivo deste estudo foi estimar a prevalência de sintomas de SAOS e examinar sua associação com características da habitação.

**TIPO DE ESTUDO E LOCAL:**

Estudo transversal em faculdade de medicina.

**MÉTODOS:**

Dados demográficos, habitacionais e de medidas corporais sobre 5.545 indivíduos de 16 anos ou mais, de diversas raças, foram selecionados do National Health and Nutrition Examination Survey. Analisamos a probabilidade de SAOS com base no ronco habitual combinada com sonolência diurna e/ou apneia testemunhada. Análise univariada e regressão linear múltipla foram usadas.

**RESULTADOS:**

9,8% dos homens e 6,9% das mulheres relataram sintomas sugestivos de SAOS (ronco habitual, sonolência diurna e/ou apneia). A prevalência de sintomas em homens e mulheres, respectivamente, foi: ronco frequente 35,1%, 22,3%, sonolência excessiva diurna 6,4%, 3,4% e apneia frequente 14,9%, 20,6%. Através de regressão linear múltipla, sintomas de SAOS foram relacionados com gênero, idade, índice de massa corpórea (IMC), estado civil e educação. Das características da habitação, mofo ou cheiro de mofo e animais de estimação no ambiente foram associados com alta probabilidade de SAOS.

**CONCLUSÃO:**

Sintomas de SAOS foram mais prevalentes do que nos países em desenvolvimento. O meio ambiente foi um fator de risco importante, porém é mais fácil controlar e manejar fatores ambientais do que outras variáveis, como IMC ou *status* socioeconômico.

## INTRODUCTION

Obstructive sleep apnea syndrome (OSAS) is a considerable issue in public health. It is a highly prevalent disorder among middle-aged adults^1^ and is independently associated with certain risk factors. It is also related to many medical conditions such as hypertension, cardiovascular morbidity and mortality, coronary insufficiency, stroke, type 2 diabetes and pulmonary disease.^2^^,^^3^^,^^4^^,^^5^ Untreated OSAS can cause road accidents,^1^ loss of work productivity,^5^ occupational injuries^1^ and even sudden death.^4^ Therefore, this disorder may lead to a huge multilateral problem unless proper control and management is implemented. The prevalence of obstructive sleep apnea among adults in the Western world ranges from 3% to 28%,^5^ and in the United States of America (USA), the prevalence is currently estimated to be 5% to 10%.^6^^,^^7^

The prevalence of OSAS in some studies based on various questionnaires has been reported as follows: based on the Berlin Criteria, in New Zealand (2009) 2.8%,^2^ Iran (2011) 4.9%^8^ and USA (2006) 26%;^5^ based on a self-reported questionnaire, in France (2007) 3.5%^1^ and Hong Kong (2001) 2.1%;^9^ and based on the Epworth Sleepiness Scale (ESS), in Nigeria (2008) 1.2%^10^ and India (2004) 3.6%.^11^ It is noteworthy that in some regions only 10% of the population has been adequately screened for appropriate diagnosis.^4^ Discrepancies in observed prevalence and underreporting may be due to non-standardized definitions and variation between diagnostic methods.^1^

OSAS screening, diagnosis and treatment entails some challenges. Polysomnography or respiratory polygraphy is a precise method for diagnosing OSAS, but this method has its disadvantages, such as expensiveness, inaccessibility and difficulty to perform. Thus, in some cases, these disorders are not diagnosed and only a few cases are properly treated.^1^ Another diagnostic method is to screen by means of a questionnaire based on three symptoms: reported habitual snoring, daytime sleepiness and witnessed apnea.

Early detection of OSAS not only reduces the morbidity risk but also leads to significant reduction in the cost of care for other conditions.^2^^,^^10^ Many studies have been published in relation to weight and demographic variables, which all have strong relationships with OSAS.^1^^,^^2^^,^^3^^,^^8^^,^^11^^,^^12^^,^^13^

Information on environmental factors affecting this outcome is unavailable. Housing characteristics are an important environmental variable; adverse conditions are preventable and can be dealt with cost-effectively, and thus may be a determinant in decreasing the burden of disease.

## OBJECTIVE

The purpose of this study was to assess the prevalence of symptoms of OSAS in a population in the USA and analyze housing characteristic risks in relation to demographic and body mass index (BMI) variables, using the National Health and Nutrition Examination Survey (NHANES) Sleep Disorders Questionnaire and dataset.^14^

## METHODS

We used the NHANES dataset (2005 to 2006), publicly available from: www.cdc.gov/nchs/nhanes.htm. NHANES was a major program that was implemented by the United States National Center for Health Statistics (NCHS). NCHS is part of the Centers for Disease Control and Prevention (CDC) and is responsible for producing vital and health statistics for the United States. NHANES was conducted in all 50 states of the USA.

NHANES data were not obtained using a simple random sample. Rather, a complex, multistage, probability sampling design was used to select participants such that they would be representative of the civilian of the civilian, non-institutionalized US population. Oversampling of certain population subgroups was done to increase the reliability and precision of the health status indicator estimates for these groups. The NCHS used four questionnaires: demographic variables, housing characteristics, body measurements and OSAS. After the datasets for demographic variables, housing characteristics, OSAS and body measurements had been merged, inconsistencies relating to 594 sequence numbers led these individuals to be excluded from the study. Thus, 5,545 individuals aged 16 years and over were selected, from several racial groups in the United States: Mexican American, Other Hispanic, Non-Hispanic White, Non-Hispanic Black and Other Race Including Multiracial.

### Demographic variables

The population for the demographics questionnaire was interviewed directly in the subjects’ homes and a proxy was provided for individuals who could not answer the questions themselves. The variables selected for this evaluation included age, gender, marital status, education status, pregnancy status, household size and family size.

### Housing characteristics

One study participant in each family responded for the entire family and these responses were released for all members of the same family. The housing characteristics provided family-level interview data on the type of home, number of apartments in the building, age of home, number of rooms in home, time lived in home, whether home was owned or rented, water source and treatment, and allergy component-related questions about the presence of furry animals.

### Sleeping characteristics

This section included questions on sleep habits and disorders. A subscale of eight questions, relating to general productivity, from the Functional Outcomes of Sleep Questionnaire, was also included.^14^ Variables pertaining to OSAS were selected in order to analyze the probability of OSAS based on habitual snoring (often or almost every night), combined with daytime sleepiness (often or almost always) and/or witnessed apnea (often or almost every night, as confirmed by self-reports).

### BODY MASS INDEX (BMI)

All survey participants were eligible for the body measurement component. There were no medical, safety or other exclusions for the body measurement protocol. The body measurement data were collected by trained health technicians, who were accompanied by a recorder during each body measurement examination. The health technicians used their discretion to obtain as many measurements as practical for individuals who were using a wheelchair. Body weight data for individuals who had had limb amputations and also those from pregnant women were excluded from the analysis. Height was measured using a Seca electronic stadiometer, in an upright standing position, with head and heels against the stadiometer before taking the height, unless this position was anatomically impossible. Before the measurement, the participants took a deep breath and held it while the headboard was positioned. If the individuals were unable to stand with the head and heels against the stadiometer, the trunk needed to be vertical above the waist and the arms and shoulders needed to be relaxed.^14^

Weight was measured on a Toledo digital scale, in pounds with automatic conversion to kilograms. The participants were weighed in their underwear. They were instructed to stand still at the center of the scale platform facing the recorder, with their hands at their sides, and to look straight ahead. After they had been properly positioned and the digital readout was stable, the recorder clicked on the capture button on the screen. The weight, length and height measurements were entered directly into the computer system by clicking on the “Get” buttons.^14^ In accordance with the World Health Organization (WHO) definitions, BMI (kg/m^2^) less than 18.5 was considered to be underweight, BMI greater than 25 was considered to be overweight and greater than 30 was considered to be obese.^15^

### Analysis

After assessment to check that the data presented normal distribution, all the continuous variables were summarized in terms of mean ± standard deviation (SD) and categorical variables were expressed as percentages. All significance tests were two-sided, and P values less than 0.05 were considered to be statistically significant. Univariate and multiple linear regression were used for the analysis. Variables showing associations with P values less than 0.10 in univariate analyses were considered to be candidate risk factors to be used in multiple analysis.

## RESULTS

The participants’ mean age was 41.8 ± 20 years. Among this sample of individuals ³ 16 years of age from the USA, 2,826 (51%) were women and 2,719 were men (49%) and consisted of the following: Mexican Americans (1,248 cases, 22.5%), Other Hispanics (174 cases, 3.1%), Non-Hispanic Whites (2,468 cases, 44.5%), Non-Hispanic Blacks (1,428 cases, 22.5%) and Other Race Including Multiracial (227 cases, 4.1%). The frequency distribution among the study participants is shown in Table 1.

**Table 1.** Distribution of participants according to demographic characteristics

Name of variable	Co-factors	Mexican American n = 1248	Other Hispanic n = 174	Non-Hispanic (White) n = 2468	Non-Hispanic (Black) n = 1428	Other n = 227
Age (years), mean (standard deviation)		36 (17.8)	36 (16.7)	46.9 (20.6)	39.5 (19.5)	37.9 (16.9)
Men, n (%)		608 (47.7)	81 (46.6)	1224 (49.6)	712 (49.9)	94 (41.4)
Body mass index (kg/m^2^), n (%)	< 18.5	28 (2.3)	4 (2.3)	68 (2.8)	32 (2.3)	10 (4.6)
	18.5-4.9	366 (30)	61 (35.3)	829 (34.6)	427 (30.7)	95 (43.6)
	25-29.9	449 (36.8)	57 (32.9)	759 (31.7)	377 (27.1)	57 (26.1)
	³ 30	376 (30.8)	51 (29.5)	738 (30.8)	555 (39.9)	56 (25.7)
	missing	29	1	74	37	9
Education status* n (%)	1	199 (16)	20 (11.6)	159 (6.5)	223 (15.7)	29 (12.9)
	2	31 (2.5)	3 (1.7)	12 (0.5)	12 (0.8)	2 (0.9)
	3	356 (28.6)	37 (21.4)	102 (4.2)	58 (4.1)	19 (8.4)
	4	189 (15.2)	33 (19.1)	223 (9.1)	233 (16.4)	8 (3.6)
	5	234 (18.8)	22 (12.7)	633 (25.8)	324 (22.9)	48 (21.3)
	6	190 (15.3)	41 (23.7)	721 (29.4)	409 (28.9)	52 (23.1)
	7	46 (3.7)	17 (9.8)	602 (24.6)	158 (11.2)	67 (29.8)
	missing	3	1	16	11	2
Marital status n (%)^†^	1	568 (45.5)	82 (47.4)	1316 (53.4)	419 (29.3)	105 (46.3)
	2	49 (3.9)	4 (2.3)	174 (7.1)	86 (6.0)	10 (4.4)
	3	58 (4.6)	9 (5.2)	230 (9.3)	118 (8.3)	179 (7.5)
	4	40 (3.2)	2 (1.2)	41 (1.7)	65 (4.6)	6 (2.6)
	5	417 (33.4)	57 (32.9)	530 (21.5)	629 (44.0)	71 (31.3)
	6	116 (9.3)	19 (11.0)	172 (7.0)	111 (7.8)	189(7.9)
	missing	-	1	5	-	-

*Education status. 1: school student for more than 4 years without any failure in end-of-year examinations and thus no repetition of the school year; 2: school student for more than 4 years with failure in end-of-year examinations and the need to repeat the school year; 3: less than 9^th^ grade, 4: 9-11^th^ grade; 5: high school graduate; 6: university student without a certificate; and 7: university graduate.

^†^Marital status. 1: married; 2: widowed; 3: divorced; 4: separated; 5: never married; and 6: living with parent.

**Graph 1.**
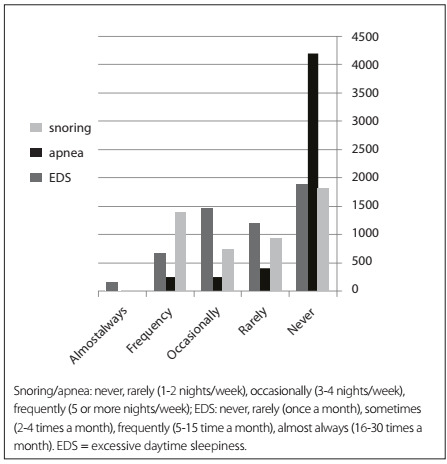
Prevalence of obstructive sleep apnea syndrome-related symptoms based on intensity

The prevalence of habitual snoring (³ 5 nights/week) was 28.7% (confidence interval, 95% CI: 27.6-29.8); habitual apnea (³ 5 nights/week) was 4.8% (95% CI: 4.6-5.0) and habitual excessive daytime sleepiness (EDS) (³ 5 times a month) was 17.9% (95% CI: 17.15-18.65). The prevalences of habitual snoring, apnea and EDS were 35.1% (849 subjects; 95% CI: 33.2-36.8), 6.4% (160 subjects; 95% CI: 6.0-6.8) and 14.9% (405 cases; 95% CI: 19.4-20.6) among the men and 22.3% (539 subjects; 95% CI: 20.95-23.36), 3.4% (87 subjects; 95% CI: 3.2-3.7) and 20.6% (584 subjects; 95% CI: 19.4-21.8) among the women, respectively. The OSAS symptoms had strong correlations (snoring/apnea: P < 0.0001 and ß = 0.39; snoring/EDS: P < 0.0001 and ß = 0.09; and EDS/apnea: P < 0.001 and ß = 0.12). The estimated prevalence of OSAS was 8.3% (463 subjects; 95% CI: 7.6-9.0). The prevalence of symptoms associated with OSAS is shown in Graph 1. The prevalence of symptoms associated with OSAS according to house characteristics is presented in Table 2.

**Table 2.** Prevalence of the symptoms of obstructive sleep apnea syndrome (OSAS) according to house characteristics

Housing characteristics	Sleep disorders	Snoring	Apnea	EDS
		N (%)		
Type of house	Mobile home or trailer	185 (50)	47 (12.3)	85 (20.5)
	One-family house, isolated	1350 (45)	315 (10)	586 (17.1)
	One-family house*	199 (40.9)	37 (7.2)	98 (17.7)
	Apartment	358 (41.9)	92 (9.9)	188 (18.8)
	Dormitory	27 (32.1)	5 (5.9)	27 (29.7)
How many apartments in the building?	1 or 2	24 (42.1)	5 (8.3)	10 (14.7)
	3 or 4	49 (38.0)	20 (13.2)	31 (19.1)
	5 to 9	104 (42.3)	27 (10.5)	45 (16.7)
	10 to 19	97 (44.1)	23 (9.7)	49 (19.8)
	20 to 49	36 (39.1)	3 (3.1)	20 (18.7)
	50 or more	38 (42.7)	13 (13.1)	27 (22.9)
When was home built?	1990 to present	79 (45.7)	119 (11)	203 (17.5)
	1978 to 1989	316 (42.8)	82 (10.7)	153 (18.7)
	1960 to 1977	331 (46.8)	71 (9.7)	160 (20.3)
	1950 to 1959	199 (47.7)	52 (11.6)	79 (16.3)
	1940 to 1949	240 (45.3)	27 (9.2)	52 (16.4)
	Before 1940	125 (44.5)	51 (9.5)	127 (21.4)
Rented or owned	Owned	1418 (45.5)	336 (10.4)	597 (17)
	Rented	653 (41.9)	152 (9)	348 (19.1)
	Other	48 (42.9)	8 (6.5)	38 (27.5)
Source of tap water	Company	1761 (43.8)	404 (9.5)	809 (17.5)
	Well	328 (47.1)	82 (11.6)	152 (19.8)
	Other	15 (46.9)	6 (19.4)	7 (20)
Water treatment devices	Yes	549 (45.1)	130 (10.3)	214 (15.6)
	No	1559 (44)	360 (9.6)	760 (18.7)
Mildew or musty smell	Yes	324 (43.7)	81 (10.3)	188 (21.9)
	No	1789 (44.3)	412 (9.7)	790 (17.1)
Cockroaches seen in home	Yes	389 (42.5)	85 (8.6)	195 (18.4)
	No	1730 (44.6)	411 (10.1)	788 (17.8)
Living or spending time with animals	Yes	916 (47.3)	210 (10.4)	449 (20.7)
	No	1203 (42.1)	286 (9.4)	535 (16.1)
Dog in house now	Yes	662 (47.2)	157 (10.8)	336 (21.4)
Cat in house now	Yes	408 (46.9)	98 (10.9)	202 (20.9)
Small furry animal in house now	Yes	69 (46.9)	12 (8.1)	42 (26.1)
Population density in house†	> 1	1767 (45.2)	434 (10.6)	812 (82.9)
	1-2	322 (40.9)	58 (6.7)	150 (16.1)
	2	21 (32.8)	2 (3)	16 (23.9)
	> 2	7 (41.2)	2 (10)	1 (5)
How many years has family lived in home?	Less than one year	402 (41.8)	96 (9.5)	193 (17.9)
	1-2 years	375 (42.2)	92 (9.6)	201 (19.4)
	3-5 years	371 (43.4)	82 (9.2)	169 (17.4)
	6-10 years	326 (47.1)	66 (9.1)	134 (17)
	More than 10 years	645 (46.3)	160 (11)	286 (17.8)

EDS = excessive daytime sleepiness; *Attached to one or more houses; †Total number of people in the household divided by the number of rooms.

In order to investigate variables relating to OSAS, univariate and multiple linear regression tests were performed. Variables with more than two categories were analyzed in the form of a dummy variable. These variables included race, education status, marital status and type of house.

In the univariate linear regression, BMI, age, gender, race, education status, marital status, type of house, age of home, rented or owned home, source of water, presence of mildew or musty smell, living or spending time with pets and ratio of population density in the home were variables that had significant relationships with OSAS.

The details the of analysis were as follows: the probability of OSAS increased linearly with increasing age and BMI respectively (ß = 0.087, P < 0.0001; ß = 0.026, P < 0.0001); the probability among the women was less than among the men (ß = -0.026, P = 0.0008); the probability among the Mexicans was greater than among other races (ß = 0.43, P < 0.0001); the probability was less among Non-Hispanic Blacks (ß = - 0.43, P < 0.0001).

The probability of OSAS among school students (16-20 years) without delay (i.e. students who never failed in the end-of-year examinations and thus never had to repeat the school year) was less than among others (ß = 1.43, P < 0.0001), while university students without a certificate and university graduates had higher probability of disorders (ß = -0.36, P < 0.0001). Regarding marital status, married (ß = -0.3, P < 0.0001), widowed (ß = -0.59, P < 0.0001, 95% CI: -0.94-0.23), divorced (ß = -0.94, P < 0.0001) and separated participants (ß = -1.19, P < 0.0001) presented higher probability of OSAS, while participants who had never married (ß =1.03, P < 0.0001) had lower probability of OSAS.

From examining the associations shown between the home environment and the disorder, the following results could be inferred: living in a mobile home or trailer produced a higher probability of OSAS than shown by other types of home (ß = 0.79, P = 0.005). In addition, a direct linear relationship was found between duration of living in a house and probability of the disease.

Homes with a well as the water source (rather than from a water supply company) and those with mildew or a musty smell had higher probability of disorders (ß = 0.79, P = 0.005) and (ß = -0.34, P = 0.003).

Living with pets was also associated with greater possibility of OSAS (ß = - 0.33, P < 0.0001), but population density presented an inverse relationship with OSAS (ß = -0.63, P < 0.0001). The variables of number of apartments in the building, home age and having cockroaches in the home did not show any association with OSAS.

In multiple linear regression analysis, the variables of age, gender, BMI, education status, marital status, mildew or musty smell, and animals living or spending time in the home presented significant relationships with OSAS. The test results are shown in Table 3.

**Table 3.** Analytical statistics on the status of obstructive sleep apnea syndrome, from multiple linear regression test

Variables*	ß	SE	P-value
Body mass index (kg/m^2†^)	0.08	0.007	0.0001
Age	0.01	0.004	0.004
Gender: 1: male; 2: female	-0.36	0.095	0.0001
Race^‡^ (dummy)			
Race 1	0.39	0.22	0.077
Race 3	0.11	0.196	0.56
Race 4	0.22	0.21	0.29
Education status^†^ (dummy)			
Education status 1	0.52	0.21	0.012
Education status 2	0.59	0.597	0.33
Education status 3	-0.19	0.21	0.37
Education status 5	-0.17	0.131	0.20
Education status 6	-0.16	0.121	0.20
Marital status^§^ (dummy)			
Marital status 1	0.22	0.21	0.29
Marital status 2	-0.12	0.291	0.68
Marital status 3	-0.34	0.26	0.19
Marital status 4	-0.93	0.37	0.01
Marital status 5	0.27	0.23	0.22
Type of house^||^ (dummy)			
Type of house 1	-0.13	0.18	0.48
Type of house 2	-0.22	0.17	0.19
When was home built?	0.02	0.03	0.40
How many years has family lived in home?	-0.03	0.04	0.50
Water source: 1: Company 2: Well	0.11	0.131	0.39
Has home had mildew or a musty smell? 1: Yes 2: No	-0.41	0.13	0.002
Have you seen cockroaches in your home? 1: Yes 2: No	-0.09	0.14	0.516
Do animals live or spend time in home? 1: Yes 2: No	-0.31	0.10	0.002
Ratio of population density	-0.10	0.15	0.49

*Variables with P < 0.1 are entered in the model; ^†^Education status 1: school student for more than 4 years without any failure in end-of-year examinations and thus no repetition of the school year; 2: school student for more than 4 years with failure in end-of-year examinations and the need to repeat the school year; 3: less than 9^th^ grade, 4: 9-11 grade; 5: high school graduate; 6: university student without certificate; 7: university graduate; ^‡^Race: 1: Mexican American; 2: other Hispanic; 3: non-Hispanic (white); 4: other; ^§^Marital status: 1: married; 2: widowed; 3: divorced; 4: separated; 5: never married; 6: living with parent; ^||^Type of house: 1: mobile home or trailer; 2: one-family house, detached from any other house; 3: one-family house, attached to one or more houses; 4: apartment; 5: dormitory.

SE = standard error.

## DISCUSSION

To the best of our knowledge, this study is the first broad study on data from the USA to contain valuable information about sleep disorders and risk factors relating to housing characteristics in the presence of important variables like weight and demographic variables. The prevalence of symptoms of OSAS was 8.3%.

We compared several studies conducted among adult in different countries using questionnaire instruments. The prevalence of OSAS was 1% in Nigeria^10^ (0.5% among women and 1.9% among men), 3.6% in India,^16^ 3.1% in Hong Kong^9^ and 5% in Iran;^8^ however, it was 7.4% in France^1^ and 26% in the USA.^5^ OSAS in the United States, like in other Western countries, is more common than in developing countries. Obesity and aging are probably crucial factors in the United States, and the rising trend of these two factors requires more attention to this context.^17^^,^^18^

Although the prevalence of risky apnea in the present study was similar to findings in Pakistan (10-12%),^19^ it was more common in Malaysia (15.2%).^20^ On the other hand, rates if 3.5% and 6.1% were reported in France and Turkey, respectively.^1^^,^^12^ Nevertheless, it should be noted that 90% of individuals with sleep apnea are undiagnosed.^21^

The prevalence of habitual snoring was similar to findings from studies conducted in the USA (46%) and Malaysia (47.3%).^5^^,^^20^ The prevalence observed in our study was higher than in the following other regions: Nigeria 31.6%,^10^ São Paulo 31%^22^ and France 22%.^1^

The rate of excessive daytime sleepiness in the present study (15%) was similar to findings from France (16%) and Malaysia (14.8%).^1^^,^^20^ On the other hand, the reported prevalence was 6.5% in the USA (2005).^5^

Consistent with a study conducted in France,^1^ habitual snoring and apnea were more prevalent among men (35.1 and 6.4%) than among women (22.3 and 3.4%). However, EDS was more prevalent among women (20.6 versus 14.9%). The prevalence of symptoms in the French study was consistently lower (61%, 7% and 24%).^1^

OSAS was less common among women, and this was similar to other studies; for instance in France, Japan, New Zealand^1^^,^^2^^,^^4^ and also in Nigeria, India, and Hong Kong.^9^^,^^10^ Epidemiological studies have confirmed that the gender ratio of OSAS is 2 to 3.1,^10^^,^^20^^,^^21^ and this ratio was 1.42 in our study. In addition to cases of more prevalent OSAS disorders among men, these differences were statistically significant in our study and some other studies^1^^,^^2^^,^^4^^,^^8^^,^^23^ although a study conducted in Iran did not reach any significant variation.^8^

BMI has been an important modifiable risk factor in relation to occurrences of OSAS.^24^^,^^25^^,^^26^ This has been confirmed in several studies.^1^^,^^8^^,^^10^^,^^11^^,^^12^^,^^13^^,^^17^ For example, obese individuals presented a risk of OSAS that was 10 times higher in the study by Salvador et al.^27^ There was also a strong relationship between these in the presence of confounding variables. On the basis of several studies, increased body weight can alter the normal upper airway mechanics during sleep through a variety of distinct mechanisms.^28^

There was a direct trend between age and OSAS in the present study. These results were consistent with reports from different countries^1^^,^^5^ in which some of the results were analyzed from multiple tests, like in the present study.^4^^,^^8^ Thus, the effect of age-related chronic diseases on OSAS needs to be investigated.

Education was another variable significantly associated with OSAS, with regard to confounding variables. It was found that high school students who never failed in the end-of-year examinations and thus never had to repeat the school year presented lower probability of OSAS. There was a direct association between education status and OSAS.^8^

Participants whose marital status was “separated” showed less possibility of OSAS than shown by other marital statuses. In a report from Nigeria, being married was a risk factor for OSAS.^10^

Although Mexican Americans presented a high risk in univariate regression, multiple regressions did not support this result. In other studies, Asian, African-American and Hispanic racial groups presented higher risk.^13^

One main goal in the present study was to investigate the association between housing characteristics and OSAS. Although the home environment has been reported as a factor in people’s health,^29^ its role in relation to sleep status had not been particularly studied.

Although the variables of type of house, population density in the home, water source and the age of the house were significant in the test, their relationship did not maintain significance in the multiple model.

An odor of mildew or a musty smell and living or spending time with animals had strong relationships with OSAS both in univariate and in multiple linear regression.

Our results are new in terms of exposure factors associated with OSAS. Whether the mechanisms through which housing characteristics correlate with OSAS are pathological mechanisms (e.g. through the activity of some fungi such as *Aspergillum* in damp environments or microorganisms found in the saliva of wool or animal), or are autoimmune mechanisms (e.g. through immune reactions against allergens such as wool and animal hair), or are physiological (e.g. through affecting concentration due to moisture and the air pressure in the home environment), these can be evaluated and thus, eventually, effective management of OSAS may be achieved.

Housing characteristics should be taken into account for public health purposes for better management of OSAS in the US, as well as in clinical decision-making relating to this syndrome.

## CONCLUSION

Symptoms of OSAS were more prevalent in the USA (8.3%) than in developing countries. Moreover, the environment was an important factor for OSAS.
